# Establishing Self-Harm Registers: The Role of Process Mapping to Improve Quality of Surveillance Data Globally

**DOI:** 10.3390/ijerph20032647

**Published:** 2023-02-01

**Authors:** Emily Bebbington, Rob Poole, Sudeep Pradeep Kumar, Anne Krayer, Murali Krishna, Peter Taylor, Keith Hawton, Rajesh Raman, Mohan Kakola, Madhu Srinivasarangan, Catherine Robinson

**Affiliations:** 1 Wrexham Academic Unit, Centre for Mental Health and Society, Bangor University, Wrexham LL13 7YP, UK; 2 Department of Emergency Medicine, Ysbyty Gwynedd, Bangor LL57 2PW, UK; 3 South Asia Self-Harm Initiative, JSS Hospital, Mysuru 570 004, India; 4 Department of Clinical Psychology, JSS Hospital, Mysuru 570 004, India; 5 Division of Psychology and Mental Health, University of Manchester, Manchester M13 9PL, UK; 6 Centre for Suicide Research, University of Oxford, Warneford Hospital, Oxford OX3 7JX, UK; 7 Department of Psychiatry, JSS Hospital, Mysuru 570 004, India; 8 Department of Plastic Surgery and Burns, Krishna Rajendra Hospital, Mysuru 570 001, India; 9 Department of Emergency Medicine, JSS Hospital, Mysuru 570 004, India; 10 Social Care and Society, School of Health Sciences, University of Manchester, Manchester M13 9PL, UK

**Keywords:** self-harm register, surveillance, process mapping, India, emergency care, innovative methodological approach

## Abstract

Self-harm registers (SHRs) are an essential means of monitoring rates of self-harm and evaluating preventative interventions, but few SHRs exist in countries with the highest burden of suicides and self-harm. Current international guidance on establishing SHRs recommends data collection from emergency departments, but this does not adequately consider differences in the provision of emergency care globally. We aim to demonstrate that process mapping can be used prior to the implementation of an SHR to understand differing hospital systems. This information can be used to determine the method by which patients meeting the SHR inclusion criteria can be most reliably identified, and how to mitigate hospital processes that may introduce selection bias into these data. We illustrate this by sharing in detail the experiences from a government hospital and non-profit hospital in south India. We followed a five-phase process mapping approach developed for healthcare settings during 2019–2020. Emergency care provided in the government hospital was accessed through casualty department triage. The non-profit hospital had an emergency department. Both hospitals had open access outpatient departments. SHR inclusion criteria overlapped with conditions requiring Indian medicolegal registration. Medicolegal registers are the most likely single point to record patients meeting the SHR inclusion criteria from multiple emergency care areas in India (e.g., emergency department/casualty, outpatients, other hospital areas), but should be cross-checked against registers of presentations to the emergency department/casualty to capture less-sick patients and misclassified cases. Process mapping is an easily reproducible method that can be used prior to the implementation of an SHR to understand differing hospital systems. This information is pivotal to choosing which hospital record systems should be used for identifying patients and to proactively reduce bias in SHR data. The method is equally applicable in low-, middle- and high-income countries.

## 1. Introduction

The surveillance of suicide attempts is an essential element of suicide prevention strategies [1,2]. The two primary methods to obtain these data are surveys of self-reported suicidal behaviour, and registers of those treated for self-harm at healthcare institutions, usually hospitals [1]. Hospital-based registers include suicide attempts and self-harm without suicidal intent (known as self-harm registers or SHRs) [1]. Self-harm is the act of self-poisoning or injury irrespective of motive [3]. The neutral term ‘self-harm’ is used because the determination of the intention to die can be difficult in the acute setting where the patient may be uncertain of their underlying motives [2]. Data from SHRs can be used by researchers, clinicians and policy makers. These data are particularly powerful when collected systematically and continuously [4]. They can provide information on emerging trends in behaviour and contributory factors, which can be used to develop preventative interventions and policy initiatives [1,2]. The SHR can then be used to monitor the effectiveness of the intervention, as has been demonstrated in the UK with the restriction of pack sizes of paracetamol and salicylates, and in Sri Lanka with household lockable pesticide storage [5,6]. SHR data can also inform clinical services through providing information on peak presentation times that may merit an adjustment in staffing, as well as local patient populations that might have additional needs (e.g., occupational, age, ethnicity) [2,4].

The Global Burden of Disease study estimates that in 2019 there were 5 million injuries due to self-harm with over 750,000 deaths [7]. Over a quarter of these injuries and deaths occurred in India despite its accounting for 18% of the global population [7,8]. Only 11% of low- and middle-income countries (LMICs) have suicide surveillance data of sufficient quality and availability for country comparisons, compared to 71% of high-income countries (HICs) [9]. The unavailability of these data limits global efforts to reduce suicide and self-harm, such as Sustainable Development Goal 3.4.2 to reduce premature mortality from suicide by a third by 2030 [10]. Previous attempted suicide or self-harm is the strongest predictor of future risk of suicide in HICs [11,12,13]. There is evidence that methods, sex distribution and patterns of repetition of self-harm differ between HICs and LMICs, particularly in South Asia [13,14]. There is also a physical, economic and psychosocial burden associated with self-harm [15,16,17]. These factors support the establishment and maintenance of SHRs. SHRs are unevenly distributed across the world, being strongly concentrated in HICs, but most global suicides occur in LMICs [9,18]. Even in HICs, there are only a small number of SHRs [18]. In establishing SHRs, it is essential that they are of high quality. Greater focus is needed on developing SHRs in settings that currently lack robust surveillance systems to ensure that relevant data are available to inform local interventions and evaluate policy initiatives.

The WHO Practice Manual for SHRs encourages countries to establish registers of hospital presentations of self-harm at all levels (national, subnational, regional or local), capturing and recording as many cases as possible [2]. It states that “Most hospital presentations will occur through the emergency department, but systems should be put into place to check records of all presentations to the hospital” (p. 19). This raises two issues that may lead to cases being missed by the SHR. Firstly, whilst emergency departments are ubiquitous in HICs, many LMICs have fragmented emergency care provision with few emergency departments [19,20]. Second, the manual gives little guidance on how to ensure that all cases of self-harm presenting to a hospital are captured in the SHR.

Other authoritative sources on SHRs also refer to data collection taking place in an emergency department, but it can be unclear what is meant by this and whether this data collection method is implementable globally [4,12,18,21,22]. In North America, Australasia and much of Europe, emergency departments are the physical space associated with the practice of emergency medicine [23]. Almost all patients presenting to hospitals in HICs following self-harm will be assessed, investigated and treated in an emergency department before hospital admission, where necessary. This represents a single entry point for patients requiring hospital emergency care and as such is a common point for data collection on self-harm presentations. Routinely collected hospital statistics may underestimate presentations of self-harm by up to 60% compared to an established SHR with a well-developed case-ascertainment strategy [24]. Emergency medicine training varies globally, with incomplete specialist provision in LMICs [19,20]. Emergency care in some LMICs is provided by doctors without specialist training, or by non-medical staff. These facilities are essentially triage systems. Patients are directed to relevant specialists earlier than in emergency departments. This is known as ‘Casualty’ in India [25]. Where emergency departments have been established recently, patients may not seek emergency care at a single hospital entry point. The routes by which patients gain care may not correspond to hospital policies, and we cannot assume that those who work in the system understand all processes completely [26]. Consequently, an SHR collecting data from a single pre-determined entry point that is not based on local processes could miss cases. Missed cases will lead to lower estimated rates of self-harm. They may also have different demographic or clinical characteristics. It is not possible to know whether cases that bypass casualty or emergency departments are different without capturing data on them. This could mean that collected data is systematically biased due to the selection of cases and is, therefore, less reliable.

Self-harm is prone to misclassification and certain types of self-harm injuries, such as self-immolation and major trauma, are particularly prone to this type of bias [1,27,28]. There is a risk that patients presenting with such injuries will not be included if there is a reliance on clinician judgement about whether the act was intentionally self-inflicted. SHRs require a case-ascertainment strategy tailored to the hospital system to ensure as many cases as possible are captured, and potential missing groups are recognised and accounted for in the reporting of the results [4].

Process mapping has been embraced as an essential technique in healthcare quality improvement to understand how a whole patient pathway works [29,30,31,32]. The technique is widely attributed to mechanical engineer Frank Gilbreth, who, in 1921, presented a method of depicting processes for any work environment in a standardised way [33,34]. Gilbreth argued that visualising every aspect of a process is essential to understand the potential impact of a change [34]. Gilbreth’s method has evolved into business process mapping, a widely used technique that aims to improve systems by understanding current processes that comprise a system, and then working to achieve improvements in bottlenecks or inefficiencies that are critically limiting the desired outcome of the system [35]. A product of the technique is a pictorial representation of the process being studied, known as a *process map*, which is analysed to identify where improvements could be made. Process mapping has been used in a wide variety of specialities and healthcare settings [36,37,38,39,40]. It has been employed in the field of suicide prevention to understand the barriers and facilitators to quality mental healthcare, inpatient suicide risk and the development of care pathways for self-harm in prison [41,42,43]. Process mapping is also advocated as a method to improve surveillance data, particularly civil registration and vital statistics (births and deaths) in LMICs [39,44]. There has been no previous description of its use to establish an SHR, but this systematic approach could be utilised to resolve the major problem of how to capture the wide variety of possible self-harm presentations across diverse emergency care systems. It is equally applicable in low-, middle- and high-income countries.

We aim to demonstrate that process mapping can be used prior to the implementation of an SHR to understand differing hospital systems, and that this information can be used to determine the method by which patients meeting the SHR inclusion criteria can be most reliably identified as well as mitigating hospital processes that may introduce selection bias into these data. We illustrate this by sharing in detail the experiences from two hospitals in south India.

## 2. Methods

### 2.1. Setting

The South Asia Self-Harm Initiative (SASHI) is a multinational research collaboration on self-harm, one part of which is to implement SHRs. Process mapping was conducted at two hospitals in Mysore, India that are prospective study sites in the SASHI SHR project. Krishna Rajendra (KR) Hospital is a tertiary, government-funded hospital. JSS Hospital is a non-profit hospital. KR and JSS hospitals both have approximately 1800 beds, include all major specialities, and are attached to medical colleges. Approximately 42% of the population of India receive inpatient medical care in government hospitals, 3% in non-profit hospitals and 55% in private hospitals [45]. The hospitals are funded differently and, therefore, were predicted to have differing patient processes that might affect SHR data collection.

### 2.2. SHR Inclusion Criteria

Eligible patients will be identified from routine records. The SASHI SHR inclusion criteria (Table 1) are purposefully broad to avoid assumptions on patient intention by data collectors, which we are aware is a sensitive issue in South Asia.

### 2.3. Approach

**Figure 1:**
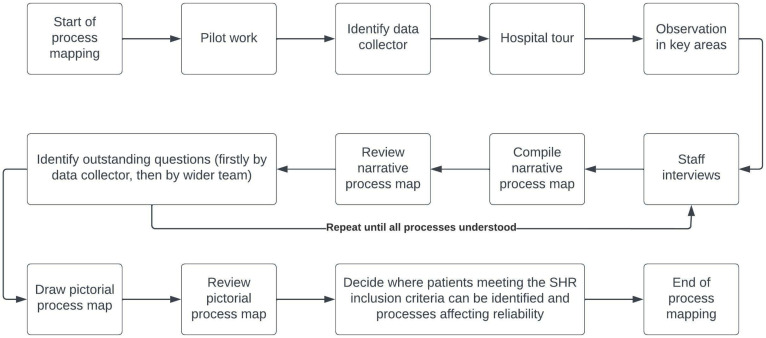
Process mapping steps completed at each hospital to determine where SHR data collection should take place and which hospital processes may affect reliability of these data.

We followed a five-phase process mapping approach developed for healthcare settings, supplemented by specific surveillance information [29,30,35]. A process mapping team was assembled from the SASHI research group, and this included clinicians and researchers from India and the UK. Clinicians included psychiatrists, a public health doctor, a psychologist and an emergency medicine doctor. The process mapping steps followed by the team are outlined in Figure 1.

### 2.4. Pilot Work

At initial meetings, it appeared that hospital processes were well understood. However, when a diagram of these processes was drawn (process map), it was unclear which hospital record systems should be used to identify patients meeting the SHR inclusion criteria. Two senior members of the group then visited the non-profit hospital to observe and discuss hospital processes with clinical staff. This showed more detailed process mapping would be feasible. It also highlighted that the clinicians only understood processes that they directly interacted with, and that uniform processes were not followed in all relevant departments. We determined a structured approach across multiple areas of the hospital was necessary to understand these processes in order to determine where patients meeting SHR inclusion criteria could be most reliably identified.

### 2.5. Data Collection

A clinician researcher gathered information during ten site visits between July 2019 and February 2020. The researcher had no prior experience of healthcare provision at either hospital, but is an experienced emergency medicine doctor in the UK so understands the principles of hospital patient flow.

Senior clinicians introduced the researcher to the hospital departments, and reassured clinical teams that permissions were in place. Initially, a tour was completed to orientate the researcher to each of the hospitals. The researcher was introduced to staff who were likely to care for patients meeting the SHR inclusion criteria, including casualty medical officers, emergency medicine doctors, intensive care doctors, plastic surgeons, general surgeons, physicians, psychiatrists, psychologists and rotational trainees. The researcher then completed a morning or an afternoon of observation in areas that were likely to receive patients meeting SHR inclusion criteria. This included casualty (government hospital), emergency department (non-profit hospital), medicolegal register office, burns unit and psychiatry department. Doctors and nurses were interviewed opportunistically during periods of observation. Experienced staff members who were patient-facing in emergency care areas were identified and asked to participate in a longer interview at a convenient time and location. These interviews often took place in the workplace, which allowed the researcher to observe them ‘in action’, thus prompting further questions.

Written field notes, photos and diagrams of the layout of key areas (e.g., hospital entrance, emergency care areas) were taken throughout. Particular attention was given to points where patients could make choices, to triage processes, to administrative procedures (e.g., registration, payment), to options for patient disposition or discharge and documentation. These details were investigated for each patient group meeting the SHR inclusion criteria.

### 2.6. Data Analysis

Interviews were reviewed for commonalities. The notes, photos and diagrams were compiled into a working document that we termed a *narrative process map*. This was analysed by the researcher who conducted the interviews for inconsistencies and to identify processes that had not been fully understood. Repeat visits and interviews were conducted in both hospitals until all outstanding questions were answered. This required three cycles. Where possible, staff of different grades and specialities were approached in each cycle. Summaries were fed back to interviewees to check accuracy. Narrative process maps were then reviewed by the entire process mapping team to identify questions and gaps that required follow-up. Interviews were conducted with experienced staff members from both hospitals by other researchers in the process mapping team to gain a different perspective. Handwritten notes were taken during interviews. Additional information was added to the narrative process maps.

The narrative process maps were translated into pictorial process maps using Business Process Model And Notation 2.0 in Lucidchart software (Lucid Software Inc., South Jordan , UT, USA): the standard form for process diagrams, irrespective of environment [46,47]. The process maps were then reviewed by the process mapping team to identify the method by which patients meeting the SHR inclusion criteria could be most reliably identified. Processes that may affect data reliability were identified, and solutions to mitigate these processes were discussed.

## 3. Results

Processes for patients meeting the SHR inclusion criteria were different across the hospital sites. Tours and interviews conducted during data collection were iterative but were found to explore similar information at both institutions despite their differing processes (Table 2).

**Figure 2:**
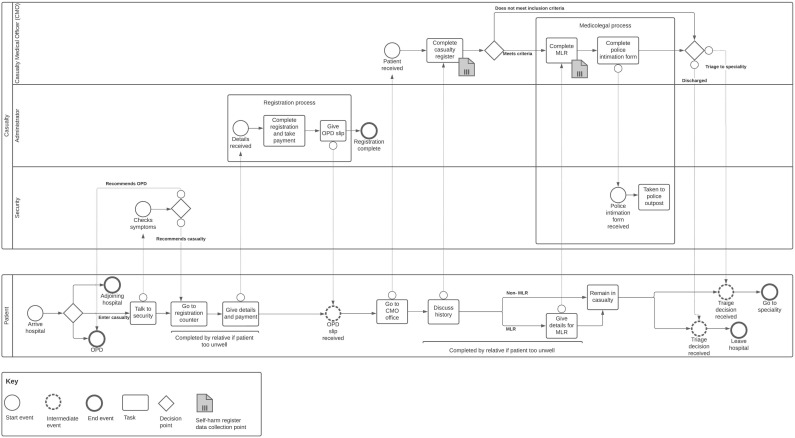
Pictorial process map of casualty in the government hospital. Grey data objects represent data source for SASHI SHR data collection. The process map has been drawn using business process model and Notation 2.0 (see key). The process map should be read from left to right. Each row (known as a lane) represents a participant in the process. Symbols (circle, triangle, square) depict events, decision points and tasks in the process. Arrows between participant lanes show the direction of flow of information for tasks occurring simultaneously. OPD = outpatient department, CMO = casualty medical officer, MLR = medicolegal register.

**Figure 3:**
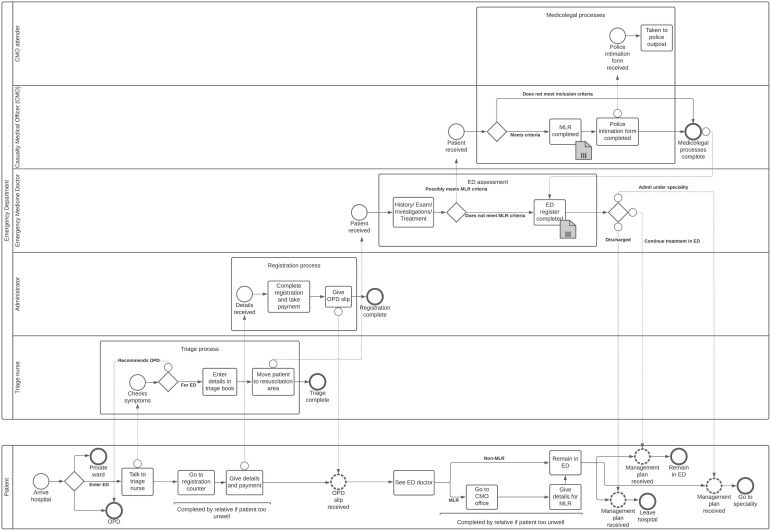
Pictorial process map of the emergency department in non-profit hospital. The same notation is used as in Figure 2.

Hospital entry points at the main emergency care areas differ significantly between the two hospitals, influencing where patients meeting the SHR inclusion criteria should be identified. The major difference is that the main emergency care area in the government hospital is casualty (Figure 2), whereas in the non-profit hospital, it is an emergency department (Figure 3).

The government hospital casualty triages patients to the appropriate speciality and completes the medicolegal processes. This includes a medicolegal register for cases that may require police investigation or legal proceedings to ascertain responsibility for injury or illness (see medicolegal processes box, Figure 2 and Figure 3). Although the Indian Mental Healthcare Act 2017 decriminalised attempted suicide, medicolegal documentation is still completed following self-harm [48,49]. The non-profit hospital records around 10 times fewer medicolegal cases than the government hospital despite having a similar number of patients presenting to the hospital (Table 3). The casualty is run by a casualty medical officer with no specialist training in emergency medicine (see casualty medical officer lane, Figure 2). At the entrance to the government hospital casualty, a security guard provides advice on where to attend (see security lane, Figure 2). Cases requiring medicolegal registration are directed to casualty.

In contrast, the non-profit hospital emergency department is run by doctors with specialist training in emergency medicine (see emergency medicine doctor lane, Figure 3). Medicolegal processes at the non-profit hospital are completed by a dedicated casualty medical officer who has no other clinical commitments and is situated across the corridor from the emergency department (see casualty medical officer lane, Figure 2). At the entrance to the non-profit hospital emergency department, patients are triaged by a senior nurse (see triage nurse lane, Figure 3). The triage algorithm at the non-profit hospital includes all SHR inclusion criteria, meaning such patients would be directed to the emergency department.

We found that SHR inclusion criteria coincide with the medicolegal register criteria that are set nationally and therefore applicable to all hospitals in India. All healthcare staff are aware of medicolegal register requirements. When a patient presents to a different part of the hospital (e.g., outpatient department, private ward) and meets medicolegal register criteria, the doctor in charge of their care is expected to report the case to the casualty medical officer for inclusion in the medicolegal register. Therefore, the medicolegal register should be the initial SHR data collection point in both hospitals.

Several processes were identified that may introduce bias into SHR data. Firstly, the patient, relative or ambulance crew chooses which hospital to attend. This is primarily a financial decision, but it may be influenced by the availability of services (e.g., ventilators), thus influencing the number of presentations to the hospitals. We have partially mitigated this through SHR data collection at multiple hospitals in the same city.

Once the patient has arrived at the hospital, they have a choice of where to seek emergency care (see patient lane arrival decision point, Figure 2 and Figure 3). At both hospitals, the patient can choose whether to attend the casualty/emergency department or the outpatient department. At the government hospital, obstetric, gynaecological and paediatric patients attend an adjoining women and children’s hospital. At the non-profit hospital, patients with medical insurance can attend a private ward directly. Patients presenting with self-harm to the outpatient department, private ward or women and children’s hospital could be missed if the healthcare professional does not refer the case to the casualty medical officer for inclusion in the medicolegal register. We are aware of two self-harm patients who presented to the outpatient department at the non-profit hospital during the study and who were not recorded in the medicolegal register: a patient whose self-laceration wounds were sutured in the outpatient department prior to discharge, and a case of partial hanging where the patient was admitted under psychiatry. Indian outpatient department clinics are extremely busy, and documentation is necessarily minimal. The outpatient department at the non-profit hospital sees 15–20 times the number of patients at the emergency department (Table 3). Because patients must be mobile and able to queue for long periods prior to being seen, only patients with minor physical injuries or self-poisonings are likely to present to the outpatient department. Patients sometimes abscond from the hospital when asked to complete medicolegal processes from the outpatient department. This is mitigated through the assistance of security staff or family escorts.

Finally, the casualty medical officer has some discretion about which cases to include in the medicolegal register, for example, self-laceration not requiring medical intervention is unlikely to be recorded. For patients who attend via casualty or the emergency department, such cases are recorded in a casualty or emergency department register of presentations but are not recorded in the medicolegal register.

Therefore, the medicolegal register should be the initial SHR data collection point in both hospitals to capture as many eligible cases as possible from the outpatient department, but case ascertainment should be optimised through cross-checking against casualty (government hospital) and emergency department (non-profit hospital) registers to capture potentially misclassified or less-severe injuries that present via casualty or the emergency department (see self-harm register data collection point symbol, Figure 2 and Figure 3).

## 4. Discussion

There is a gap in guidance on thorough case ascertainment when establishing SHRs, due to an assumption that emergency care processes are uniform internationally. We have shown how process mapping can be used to identify where patients meeting SHR inclusion criteria may be reliably identified and how to mitigate hospital processes that may introduce selection bias into these data. This innovative work demonstrates that, although two hospitals differ significantly, the same systematic method can be used to understand the relevant processes. This informs decision-making on optimal case ascertainment. Without this, SHRs identify patients from a single point of convenience and may miss significant patient groups, creating selection bias in the data. Whilst the process maps themselves will not generalise per se, the method of producing them and the need to do so will be relevant for any healthcare system wishing to establish an SHR.

A simple and reproducible method to inform case-ascertainment processes is particularly important in countries with diverse healthcare infrastructures. We have shown that two hospitals in close vicinity offer different types of emergency care. The provision of emergency care globally is moving towards the model of emergency departments run by specialist emergency medicine doctors [19,20,50]. Training programmes are not universally available, particularly in LMICs, and the lived experience of patients attending such facilities is poorly explored [51]. Repeat reference to data collection in “emergency departments” in the WHO SHR Practice Manual is unhelpful [2]. We discovered multiple pathways through which patients access care at both hospitals, with no single clinical point at which all cases of interest could be captured. Data for the SHR therefore must be collected from multiple sources and cross-referenced. This finding is likely to be applicable to other hospitals in India, though the methods could be applied globally to any hospital wishing to establish an SHR.

The existence of routes to hospital care that by-pass the hospital’s main emergency care area means that a comprehensive SHR cannot be solely based on records from those departments. We found such patients would be likely to be less severely unwell or injured and overlooking them would introduce selection bias to the data. It is conceivable that this type of bias contributes to ostensibly low rates of repetition of self-harm in South Asia [13,14,52]. Process mapping could be used in a similar way to understand the use of local primary healthcare infrastructure, and it would be an important step in assessing whether the surveillance of self-harm that occurs without hospital presentation is feasible.

The use of a systematic method to understand patient flow through healthcare facilities is particularly useful to protect surveillance when public health emergencies suddenly dictate changes to the healthcare infrastructure. For example, the COVID-19 pandemic led the Indian government to announce that all medical colleges were required to establish an emergency department staffed by emergency medicine doctors by 2022 [53]. Process mapping is well-suited to tracking changes that ensue, allowing SHR data collection to be modified and data quality maintained.

It is recognised that LMICs face greater challenges than HICs in establishing and maintaining national surveillance systems [35,51]. The establishment of SHRs at individual hospitals may provide a partial solution. Good-quality data from a few locations are more powerful than poor-quality national data. This is exemplified by the longest running SHR, the Oxford Monitoring System for Self-Harm, which has been maintained at a single hospital emergency department in the UK since 1976 and has, for example, influenced UK national policy including the regulation of sales of over-the-counter analgesics [5,54]. This supports the establishment of high-quality SHRs at individual hospitals that aim to enable local teams to evaluate interventions, outcomes and policy initiatives.

It has not been possible to estimate the extent of missing data from outpatient departments, private wards and the women and children’s hospital due to the large number of presentations to these widely spread clinical areas. These areas are more likely to receive less severely unwell or injured patients who meet the SHR inclusion criteria. Process mapping revealed that patients who meet the SHR inclusion criteria and present to locations other than casualty or the emergency department should be referred to the casualty medical officer for inclusion in the medicolegal register. Using both casualty/ED registers and the medicolegal register should mitigate some of the bias introduced by patients presenting outside of the main emergency care area. Whilst the recommendation to use the medicolegal register to capture cases is likely to only apply to South Asia, the method by which we came to this conclusion is of more general applicability.

We recommend that process mapping is completed prior to the implementation of all SHRs globally. The technique allows the detection of potential problems in their real-world context, allowing bespoke solutions to be developed. This is particularly important given the difficulty and expense associated with maintaining a clinical register and may avoid an expensive register failing to meet its goals [55,56]. Process mapping fulfils many of the same purposes as a feasibility study used prior to a randomised controlled trial, but it is more suited to the establishment of an SHR due to the innate difficulties of capturing this diverse patient group continuously and systematically [57].

The development of a ‘narrative process map’ prior to the pictorial process map is advised, particularly in international projects that include team members from different professional and national backgrounds. These allow a detailed description and explanation of each step of a process, which can be used to inform analyses. Groups wishing to establish an SHR could modify the topics detailed in Table 2 for use in semi-structured interviews completed during data collection. We also recommend that the WHO SHR Practice Manual is updated to reflect differences in emergency care provision internationally and that it includes process mapping as the preferred method to optimise case ascertainment [2].

## 5. Conclusions

Process mapping is an innovative solution to improve the data quality of newly established SHRs. It can be used to understand how patients presenting to a hospital following self-harm flow through a hospital system. This information is pivotal to choosing which hospital record systems should be used to identify patients for inclusion in an SHR, irrespective of the type of emergency care provision. It allows the identification of sources of selection bias, indicating how an SHR can be improved. The method is easily reproducible and can be used in the implementation of other registers globally.

## Figures and Tables

**Table 1:** Inclusion criteria for the SASHI self-harm register.

SASHI Self-Harm Register Inclusion Criteria
Poisoning (medication and non-medication)
Burn injury
Hanging
Drowning
Fall from height
Fall in front of train
Self-laceration
Firearm injury
Unspecified self-harm

**Table 2:** Topics and questions explored during data collection at both hospitals organised according to the typical patient journey. Full narrative process mapping documents are not presented because they include sensitive, hospital-specific information and are unlikely to be of interest to a general readership.

Topic	Specific Details Addressed in Tours and Interviews
**Overview**	Type of hospital, funding, size and specialities.
	Other types of healthcare provision available locally.
**Patient arrival**	Referral options (e.g., from other hospitals, primary care).
	Means of arrival (e.g., walk, ambulance), method of ascertaining that means (e.g., national telephone number) and associated costs.
**Point of arrival**	Departments providing emergency care at the hospital (e.g., casualty, emergency department, outpatient department).
	Method by which patients are directed to the appropriate department.
**Main emergency care area**	Consensus on the department responsible for providing immediate emergency care to newly presenting patients (e.g., casualty, emergency department).
	Triage processes for new patients.
	Patient registration and payment procedures.
	Inclusion criteria for any routinely collected registers.
	Areas of the department where patients may be treated.
**Management in the main emergency care area**	Clinician responsibilities and training.
	Whether there are any medicolegal processes.
**Medicolegal processes (if applicable)**	Clinician responsible for completing medicolegal processes (e.g., casualty medical officer).
	Patient groups requiring medicolegal processing.
	Paperwork completed for each patient (e.g., medicolegal register).
	Inclusion criteria for any routinely collected registers, and details entered into these registers.
	Clinician discretion for medicolegal processing.
	Police involvement in medicolegal processes in hospital.
	Length of time records kept.
	Payment processes.
**Disposition from main emergency care area**	Main area or specialities where patients who meet the self-harm register inclusion criteria go (if applicable).
	Inpatient admission registration and payment.
**Alternative means by which patients can access emergency care** **(e.g., outpatient department, nearby hospitals, private ward)**	Method by which patients access these services.
	Handling of medicolegal processes.
	Patient groups (e.g., priority, injury type) that could present to these areas or the main emergency care area.
	Methods by which patients can be admitted to these areas directly and bypass normal administrative routes.

**Table 3:** Number of patients presenting to each hospital and the proportion who are recorded as a medicolegal case. The breakdown of the number of patients presenting to casualty and the outpatient department of the government hospital was not available.

	Number of Patients		
	2019	2020	2021
**Government hospital (KR Hospital)**			
Outpatients (including casualty and outpatient department)	476,012	297,682	329,978
Medicolegal cases	24,404	17,969	24,029
**Non-profit hospital (JSS Hospital)**			
Outpatients (including casualty and outpatient department)	664,650	376,870	415,034
Emergency department	29,330	23,204	19,153
Outpatient department	635,320	353,666	395,881
Medicolegal cases	3969	2917	3021

## Data Availability

All data relevant to the study are included in the article.
